# Burden of mental disorders and unmet needs among street homeless people in Addis Ababa, Ethiopia

**DOI:** 10.1186/s12916-014-0138-x

**Published:** 2014-08-20

**Authors:** Abebaw Fekadu, Charlotte Hanlon, Emebet Gebre-Eyesus, Melkamu Agedew, Haddis Solomon, Solomon Teferra, Tsehaysina Gebre-Eyesus, Yonas Baheretibeb, Girmay Medhin, Teshome Shibre, Abraham Workneh, Teketel Tegegn, Alehegn Ketema, Philip Timms, Graham Thornicroft, Martin Prince

**Affiliations:** Department of Psychiatry, Addis Ababa University, College of Health Sciences, School of Medicine, Addis Ababa, Ethiopia; King’s College London, Institute of Psychiatry, Department of Psychological Medicine, Centre for Affective Disorders, London, UK; Health Services and Population Research Department, King’s College London, Institute of Psychiatry, London, UK; Amanuel Specialized Mental Hospital, Addis Ababa, Ethiopia; Department of Internal Medicine, St Paul Hospital Millennium Medical College, Addis Ababa, Ethiopia; Addis Ababa University, Aklilu Lemma Institute of Pathobiology, Addis Ababa, Ethiopia; University of Toronto, Ontario Shores Center for Mental Health Sciences, Toronto, Canada; Addis Ababa University, Ethiopian Institute of Architecture, Building Construction and City Development, Addis Ababa, Ethiopia; Mental Health Society-Ethiopia, Addis Ababa, Ethiopia; South London and Maudsley NHS Foundation Trust, London, UK

**Keywords:** Homelessness, Rooflessness, Mental illness, Severe mental disorder, Prevalence, Unmet needs, Low- and middle-income country, Ethiopia

## Abstract

**Background:**

The impact of mental disorders among homeless people is likely to be substantial in low income countries because of underdeveloped social welfare and health systems. As a first step towards advocacy and provision of care, we conducted a study to determine the burden of psychotic disorders and associated unmet needs, as well as the prevalence of mental distress, suicidality, and alcohol use disorder among homeless people in Addis Ababa, the capital of Ethiopia.

**Methods:**

A cross-sectional survey was conducted among street homeless adults. Trained community nurses screened for potential psychosis and administered standardized measures of mental distress, alcohol use disorder and suicidality. Psychiatric nurses then carried out confirmatory diagnostic interviews of psychosis and administered a locally adapted version of the Camberwell Assessment of Needs Short Appraisal Schedule.

**Results:**

We assessed 217 street homeless adults, about 90% of whom had experienced some form of mental or alcohol use disorder: 41.0% had psychosis, 60.0% had hazardous or dependent alcohol use, and 14.8% reported attempting suicide in the previous month. Homeless people with psychosis had extensive unmet needs with 80% to 100% reporting unmet needs across 26 domains. Nearly 30% had physical disability (visual and sensory impairment and impaired mobility). Only 10.0% of those with psychosis had ever received treatment for their illness. Most had lived on the streets for over 2 years, and alcohol use disorder was positively associated with chronicity of homelessness.

**Conclusion:**

Psychoses and other mental and behavioural disorders affect most people who are street homeless in Addis Ababa. Any programme to improve the condition of homeless people should include treatment for mental and alcohol use disorders. The findings have significant implications for advocacy and intervention programmes, particularly in similar low income settings.

**Electronic supplementary material:**

The online version of this article (doi:10.1186/s12916-014-0138-x) contains supplementary material, which is available to authorized users.

## Background

The problem of homelessness is not new. Ancient Greek and Roman literature makes references to homelessness; for example, the famous ascetic and cynic, Diogenes, lived in a jar in a market place [1]. More historical description of homeless people may be tracked in legislative documents from the UK dating back to the late 13th century [2]. Legislation in the early 18th century noted that for people with mental illness, ‘if the lunacy be unoffensive…(the mentally ill were) left to ramble half naked and half starved through the streets and highways, teased by the scoff and jest of all that is vulgar, ignorant and unfeeling’ [3]. The attitude of the public towards homeless people has varied between sympathy and fear [4] and desire to help and punish [2], but the old sense of stigmatisation, ostracisation and victimisation of homeless people has continued to the present day, with homeless people often dismissed as inadequate, alcohol and drug abusers, or mentally ill [5].

The history of homelessness in Africa is much less clear. Some link the onset of homelessness as a problem in Africa with the disruption of the kinship networks and loss of land ownership during colonialism: ‘As Europeans built their estates, expanded their market places, and planned their public squares, indigenous communities were left homeless and were pushed into the peripheries of urban and commercial life’ [6].

Although homelessness is a worldwide problem, estimating the number of homeless people is very difficult, as reflected by the large variations in the reported number of homeless people, with one hundred million to one billion people said to be homeless worldwide [7]. The number reported to be homeless at any given time in the UK has been between 100,000 and 400,000 [8]. This large variation reflects the difficulty of tracking homeless people as well as the variation in the definition of ‘homelessness’. Four classes of homelessness are distinguished [9]: 1) inferior or substandard housing; 2) insecure accommodation; 3) houselessness (living in institutions or short-term guest accommodation); and 4) rooflessness. Rooflessness, also known as ‘sleeping rough’ or ‘street homelessness’, is the most extreme manifestation of homelessness. Although most of the homeless people in high income countries live in sheltered accommodation, a substantial number also live on the streets; for example, over 300,000 homeless people in the USA are street homeless [10].

Generally, 25% to 50% of the homeless population are reported to have some form of mental disorder [11–14] in high income countries. This rises to about 60% among those who are street homeless. In a meta-analysis of 29 studies conducted from 1979 to 2005, the commonest disorders found among the homeless population were alcohol dependence, drug dependence and psychotic disorders, with random effects pooled prevalence estimates of 37.9%, 24.4%, and 12.7%, respectively [15]. The consequences of mental disorder among homeless people in high income countries are: 1) an increased risk of mortality from general medical causes, suicide [16–18] and drug-related causes [19]; 2) increased vulnerability of the homeless person, including violent victimisation [20] and criminality [21–23]; and 3) increased likelihood that the person spends longer periods as homeless [24].

To our knowledge, there are no studies looking explicitly at the prevalence of mental disorders among the street homeless, the needs of this group and potential solutions, or barriers to improving their care in low income countries. However, limited data are available about the experience of homelessness by the mentally ill [25]. A community-based study of people with schizophrenia reported a prevalence of 7% homelessness in Ethiopia [26]. In a 13-year retrospective study of people with schizophrenia in Nigeria, a history of homelessness was found in 4% of the sample [27]. In China, in a 10-year prospective study of people with schizophrenia, 7.8% of the sample had experienced homelessness at least once [28]. A report from India reflects only on the impact of rehabilitation of homeless women with schizophrenia [29]. There is, therefore, little to support the assumption that strong kinship may prevent homelessness in low income countries. Furthermore, when family ties have failed, there are no structured programmes or appropriate welfare systems to support homeless people with mental disorders in low income countries, thus increasing their vulnerability [27]. There are several studies in Africa looking at substance abuse, risk behaviour and trauma among street children, and these studies indicate high prevalence of substance abuse, trauma and risk behaviour among street children (see Additional file 1: Table S1).

In Ethiopia, particularly in the major cities, homelessness is a manifest problem. In Addis Ababa, for example, the city administration estimates the number of homeless individuals to be around 50,000. Although multifaceted mental health service scale-up programmes are being initiated with government backing (for example, the mental health Gap Action Programme (mhGAP) [30] and the Programme for Improving Mental health Care (PRIME) [31]), there are no tailored programmes for homeless people with mental disorders, who are very unlikely to access these integrated or specialist services because of their marginalisation and lack of family support. Homeless people with mental illness are likely to have complex physical, social and psychological needs requiring complex interventions. The primary aim of this report is to present data on the prevalence of psychotic disorders and the level of unmet need among the street homeless, with a view to informing future interventions. The secondary aim of the report is to present data on the prevalence of general mental distress, alcohol use disorder and suicidality among the street homeless. Although other mental disorders and substance abuse are important and prevalent in this population, we have focused on psychotic disorders for two reasons. First, this prioritization is in line with the priorities of the country as indicated by the National Mental Health Strategy, in which psychosis is the top priority [32]. Secondly, those with psychosis are the most vulnerable and least likely to benefit from decentralized services or programmes that are aimed at addressing the needs of homeless people.

## Methods

### Setting

The study was conducted in two districts of Lideta sub-city (districts 8 and 10) and two districts of Addis Ketema sub-city (districts 4 and 7) in Addis Ababa. Addis Ababa, the capital of Ethiopia, is divided administratively into 10 sub-cities, and in turn, each sub-city is divided into 10 to 15 districts. Addis Ketema and Lideta sub-cities are the most densely populated sub-cities in Addis Ababa, with a population density of 36,659 people/sq km and 23,395 people/sq km, respectively. These districts were selected because of their accessibility and the positive relationship the research team had developed with the district administrations during initial exploratory work.

### Design

**Figure 1:**
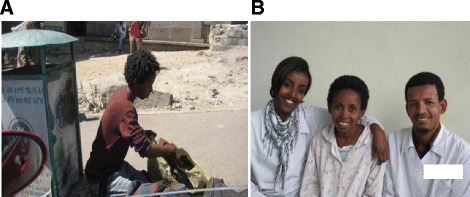
**A young woman with a 2-year history of street homelessness related to a schizophrenic illness that started while she was in college. (A)** The woman on a street in Addis Ababa packing up her belongings just before admission for treatment; **(B)** the same woman with some of the clinical team in Amanuel Hospital before she left the hospital. Written informed consent was obtained from the patient for publication of this study and any accompanying images. A copy of the written consent is available for review by the Editor of this Journal. Photo by Abebaw Fekadu.

**Figure 2:**
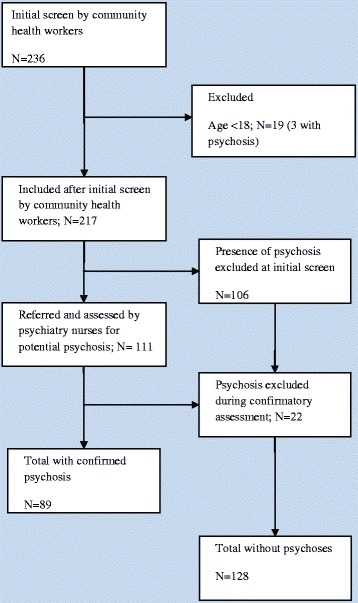
**Flow diagram of assessments for psychosis of street homeless individuals, Addis Ababa, Ethiopia.**

The study was a cross-sectional community-based study. Homelessness was equated with street homelessness (rooflessness) (Figure 1), and all individuals who had spent at least 24 hours on the street prior to the day of assessment were eligible for inclusion. A double-stage sampling design was used to identify individuals with severe mental disorders (psychotic disorders). In the first stage, individuals aged 18 years and above who were street homeless were assessed by trained community nurses. Standardized screening questionnaires (see below) were administered to all participants, and potential cases of psychosis were referred to psychiatric nurses for a confirmatory assessment (Figure 2). In the second stage, psychiatric nurses carried out diagnostic interviews to confirm the occurrence of psychosis among referred cases. If psychosis was identified, the psychiatric nurses also administered a questionnaire to evaluate the level of unmet needs of those with psychosis.

### Assessments

#### Screening assessments

Community nurses administered instruments focusing on the assessment of demographic status, potential psychosis, mental distress, suicidality and alcohol use disorder. The demographic screening tool was designed by the research team, and consisted of simple questions about the sociodemographic characteristics of the homeless person, duration of their homelessness, and whether the community nurse interviewer considered the person to have a psychotic disorder. The community nurses based their assessment of psychosis on the behavioural manifestations of psychosis among the street homeless. The nature of the training is described further below.

The community nurses also administered standard instruments for measuring mental distress (Kessler 10-item version; K10) [33], with three additional questions on suicidality and alcohol use disorder (the Alcohol Use Disorder Identification Test (AUDIT)) [34]. The K10 is a widely used tool to assess mental distress in the preceding one month [33]. Each item is rated from 1–5, from ‘none at all’ to ‘all the time’. The total score for the 10-item scale is 50, ranging from 10 to 50. Four possible categories of mental distress are specified based on the scores: 10 to 19, likely well; 20 to 24, mild disorder; 25 to 29, moderate disorder; and 30 to 50, severe disorder. Both the 10-item and 6-item versions (K10 and K6) were validated in Ethiopia, with the 10-item version showing superior validity [35]*.* We used the locally validated Amharic (the official national language of Ethiopia) version of the K10 [35].

The questions about suicide followed the style of the K10 questions, and simply asked whether the interviewee had experienced the wish to die or had suicidal thoughts, and whether the person had attempted suicide in the preceding 1 month. AUDIT [34] was developed by the WHO as a screening tool to indicate problematic consumption of alcohol in the previous 12 months in people attending primary care facilities [36]. AUDIT has 10 items, each rated on a four-point scale, giving a total score ranging from 0 to 40. Although not validated in the Ethiopian setting, AUDIT has been used in neighbouring countries [37,38]. Local alcoholic beverages were converted into standard equivalent alcohol units [39]. Four categories of use are distinguished based on AUDIT scores. A score of 0 to 7 is indicate of normal use; 8 to 15 is indicative of harmful use; 16 to 19 is indicative of hazardous use; and 20 and above indicates dependent drinking.

### Assessment for psychotic disorders

The aim of the assessment by psychiatric nurses was to establish the presence of any psychotic disorder rather than making a diagnosis of a specific psychotic disorder, as detailed diagnostic evaluation was considered *a priori* to be impossible. However, an attempt was made to make classifications in accordance to the diagnostic algorithms in the ICD-10 Classification of Mental and Behavioural Disorders [40] based on the structured observational items of the Schedules for Clinical Assessment in Neuropsychiatry (SCAN) and the items of the [41] Psychosis Screening Questionnaire (PSQ) [42]. The PSQ covers five broad categories of symptoms: hypomania, thought interference, delusions of persecution, ‘strange’ experiences and auditory hallucinations. Two or three questions are used for each symptom category: a general introductory stem question, and one or two more targeted questions for those who answer ‘yes’ to the introductory questions. The PSQ was administered by psychiatric nurses, and to screen positive on the PSQ, a participant should respond positively at least to all the questions of one of the five domains. Psychiatric nurses also documented any gross and apparent physical disability.

#### Assessment of unmet needs

The Camberwell Assessment of Needs Short Appraisal Schedule (CANSAS) was used. CANSAS is a short 22-item questionnaire, which aims to determine whether a need is present and, if present, whether it is met or unmet. The main domains of the scale include basic (for example, accommodation and food), safety (for self and others), interpersonal and family needs (for example, childcare needs), social and health-related needs. Assessments can be recorded from the perspectives of the service user, a health professional and an informal carer [43].

The CANSAS was adapted based on a series of expert consensus meetings and discussions with the developers of the original CANSAS. Three forms of adaptation were carried out. First, items considered not applicable were modified. Item 22 of the original CANSAS refers to benefits payments, which are not available in Ethiopia; we modified this to refer to benefits from family members. Second, four items considered relevant to this particular population were added. These four items related to basic needs (availability of clean water for drinking and washing, and availability of sufficient clothes and shoes), social needs (availability of close family support) and safety needs (the sense of threat perceived by the homeless individual). Thus the total number of specific needs assessed was increased from 22 to 26. Finally, these items were converted into question statements. The final version of the adapted CANSAS was approved by the developers of the original CANSAS. Psychiatric nurses administered the CANSAS once they had determined that psychosis was present. The adapted version of the CANSAS is provided (see Additional file 2: Annex 1).

### Training of assessors

The community workers were first trained to recognise the manifestations of mental disorders, and how to distinguish severe mental disorders in homeless people from behaviours that may develop in the context of chronic homelessness. This training was provided by a UK psychiatrist (PT), who leads a service for homeless people with mental illness in South London and has considerable expertise in this area. Further training of community workers and training in the use of all of the instruments was given by an Ethiopian psychiatrist (AF) with experience in training interviewers, including users of complex psychiatric instruments such as the SCAN.

### Procedure of evaluation and participant identification

Evaluation was carried out over consecutive days, including weekends. All assessments took place between approximately 06.00 and 09.00 hours. This enabled interviewers to assess homeless people before they left their sleeping sites, and to ensure that the sample did not include people who beg on the streets during the day but are not street homeless. Furthermore, after 09.00 hours, the streets become too busy to conduct interviews in a confidential manner. On the morning of the assessment, the assessors, including the coordinating psychiatrist, met at a pre-designated street corner. From there, they walked through the adjoining streets, approaching anybody who appeared to be homeless. The community nurses worked locally and often knew the homeless individuals, which also helped to determine whether they thought the individual they assessed might have psychosis. The psychiatric nurses provided supervision to the community nurses.

Both groups of assessors were supervised by a senior mental health practitioner with a Masters level training and by a senior psychiatric nurse. The overall conduct of the assessments was supervised by an Ethiopian psychiatrist. The interviews were conducted in churchyards and often on the streets, with care taken to maximise privacy. When questionnaires were incomplete, the data collectors were asked to go back and attempt to complete the questionnaires. Ten psychiatric nurse interviewers and 20 community nurses were involved in carrying out assessments. To ensure the safety of interviewers, they were accompanied by community police, who watched discreetly from a distance without interfering with the interviews. Virtually no police assistance due to threats to interviewers during the survey was required.

### Sample size

A sample size of 95 would allow us to test the hypothesis that the prevalence of psychosis among homeless people is 30% [11] with a 95% confidence interval, 90% power, margin of error of 15% and a non-response rate of 10%. We oversampled in order to achieve the secondary objectives of determining the prevalence of mental distress, suicidality and alcohol use disorder.

### Data management and analysis

Data were double-entered using Epidata, v 3.1 (The EpiData Association, Odense, Denmark) and exported to the IBM SPSS, v 20 (IBM Corp., NY) for analysis. The analyses were primarily descriptive, focusing on frequencies and percentages of outcomes of interest. In determining prevalence, the standard cut-off scores of the K10 and the AUDIT were used as described above. Denominators for frequencies and percentages were based on the number of individuals with data available on a particular item. Comparative analysis was used to look at factors indicative of chronicity of homelessness, and to compare those with and without psychoses. We used complete case analysis to deal with missing data in the few comparisons we conducted.

### Ethical considerations

The study was led by Amanuel Hospital and was a collaborative project with Addis Ababa University, the Mental Health Society-Ethiopia and King’s Health Partners. Ethical approval was obtained from King’s College London Research Ethics Committee (PNM/10/11-164) and the Ethical Review Committee of Amanuel Hospital (AM/147/5/1932). In all cases, informed consent was sought after adequate information about, and the potential benefits and risks of the study had been provided. In circumstances where mental illness was impairing a person's capacity to consent, we sought permission from a guardian or a community representative. Given the lack of knowledge on setting up services for homeless individuals with psychosis, it was considered crucial to allow as many people with psychosis as possible to participate in the study. It was particularly important for the study that those who lacked capacity to consent were still able to participate because these were likely to be ill and to be the most vulnerable. Therefore, when it was certain that a person was unable to consent because of lack of capacity due to mental illness, and despite attempts to establish rapport, we sought permission from a guardian or a representative of the community or the district. When interviews were conducted under such circumstances, this occurred only if the person being interviewed was not actively refusing or resisting. Assessments were conducted in private except when the interviewees preferred to be interviewed in the company of their friends. The research team facilitated admission, in collaboration with the district administration and Amanuel Hospital, for those considered at immediate risk, including admission to a rehabilitation unit. The research team also referred any child at high risk of harm and neglect to an adoption agency, which was done with the mother’s full consent. Informed consent was obtained from the person shown in Figure 1 for the publication of the photographs.

## Results

### General characteristics

In total, 217 homeless people were evaluated. Most were men (n = 195; 90.3%). Of those with psychotic disorder (n = 89), about 90% were men (Table 1; Table 2). The mean ± SD age of participants was 32.6 ± 14.0 years, ranging from 18 to 78 years. Those with psychosis were significantly older (37.1 ± 13.7 years) than those without psychosis (29.1 ± 13.2 years) (Table 3). Most participants with psychotic disorder were chronically homeless, with over two-thirds having been homeless for 2 years or longer; just 16.7% were homeless for less than 6 months (Table 4). Most had no formal education, and nearly half had never been employed. Most of the homeless participants had migrated into Addis Ababa from elsewhere in Ethiopia (76.5%). Moreover, most with psychosis who responded said they did not have a place to which to return (55.0%), and that they had not seen their family since becoming homeless (58.3%). Only 6.6% had visited their family in the previous 12 months.

**Table 1:** **Selected demographic characteristics of homeless people with psychoses, Addis Ababa, Ethiopia**

**Characteristic**	**Response categories**	**Number**	**Percent** ^**a**^
Gender (n = 89)	Male	80	89.9
	Female	9	10.1
Age (n = 85)	18-24	12	14.1
	25 to 34	26	30.6
	35 and above	47	55.3
Marital status (n = 59)	Single	52	88.1
	Divorced	5	8.5
	Separated	2	3.4
Children (n = 49)	No	39	89.9
	Yes	10	20.4
Education (n = 60)	Not literate	33	55.0
	Primary	20	33.3
	Secondary	5	8.3
	Post secondary	2	3.3
Ever employed (n = 59)	No	28	47.5
	Yes	31	52.5
Address before being homeless	Addis Ababa	12	23.1
	Outside Addis Ababa	40	76.9

**Table 2:** **Prevalence of current mental and alcohol use disorder among homeless people, Addis Ababa, Ethiopia**

**Condition**	**Severity**	**Number**	**Percent** ^**a**^
Psychosis	Any under	89	41.0
Mental distress N = 121	Any mental distress (excluding psychosis)	90	74.4
	Mild	25	20.7
	Moderate	24	19.8
	Severe	41	33.9
Alcohol use disorder N = 188	Hazardous use	38	20.2
	Harmful use	21	11.2
	Dependent use	53	28.2
Suicidality	Frequent/persistent death wish (n = 184)	77	41.8
	Frequent/persistent suicidal ideation (n = 184)	40	21.7
	Suicide attempt (n = 209)^b^	31	14.8

^a^Percentage for psychosis based on the total screened adults (n = 217) as denominator; for other percentages, the denominators are provided under specific categories. The denominators represent the number of individuals with complete information for a particular variable or outcome of interest.

^b^Supplemented by information from informants.

**Table 3:** **Comparison of homeless individuals with and without psychosis on selected demographic and clinical characteristics**

**Characteristics**	**Categories**	**Non-psychosis**		**Psychosis**		***P*** **-value**
		**n**	**%**	**n**	**%**	
Gender	Male	115	89.8	80	89.9	1.000
	Female	13	10.2	9	10.1	
Age	<25	12	14.1	54	45.8	<0.001
	25 to34	26	30.6	37	31.4	
	≥35	47	55.3	27	22.9	
Duration of homelessness	<6 months	4	5.5	14	12.5	0.051
	6 to 12 months	6	8.2	15	13.4	
	1 to 2 years	5	6.8	11	9.8	
	2 to 5 years	18	24.7	27	24.1	
	>5 years	40	54.8	45	40.2	
Death wish (past month)	None or minimal	31	49.2	76	62.3	0.088
	Frequent or persistent	32	50.8	46	37.7	
Suicidal thoughts (past month)	None or minimal	44	69.8	100	82.0	0.060
	Frequent or persistent	19	30.2	22	18.0	
Suicide attempt (past month)	None	43	69.4	104	86.0	0.008
	At least once	19	30.6	17	14.0	
Alcohol use	Healthy use	16	25	61	48.8	<0.001
	Hazardous use	9	14.1	29	23.2	
	Harmful use	9	14.1	12	9.6	
	Dependent use	30	46.9	23	18.4

**Table 4:** **Some selected characteristics of homelessness people with psychotic disorder (n = 89)**

**Characteristics**	**Response categories**	**Number**	**Percent**
History of homelessness (n = 57)	No	34	59.6
	Yes	23	40.4
Duration of homelessness (n = 73)	<6 months	4	5.5
	6 to 12 months	6	8.2
	1 to 2 years	5	6.8
	2 to 5 years	18	24.7
	>5 years	40	54.8
Have place to return to (n = 57)	No	33	55.0
	Yes	27	45.0
Last visit with family (n =60)	1 to 6 months ago	2	3.3
	7 to 12 months ago	2	3.3
	>12 months ago	21	35.0
	Never seen them since leaving	35	58.3

### Prevalence of mental disorders

**Figure 3:**
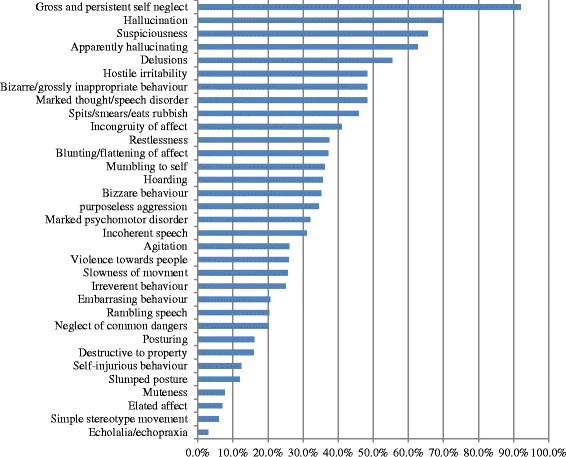
**Profile of symptoms and observed behaviours of street homeless with psychosis.**

Details are presented in Table 2. About nine in ten individuals had some form of mental or alcohol use disorder, and a substantial proportion (41.0%; n = 89/217) had psychotic disorders. Most of those with psychosis had schizophrenia (88%; n = 79), while the rest had either nonorganic psychotic disorders (9.0%; n = 8) or psychosis related to bipolar disorder (2.2%; n = 2). Excluding those with psychosis, mental distress measured with K10 was the commonest condition (74.4%) among those without psychosis. Problematic alcohol use was found in 60.0% of the sample that responded to the AUDIT questions (n = 181). Most of the alcohol use disorder was co-morbid with other mental disorders: in 74.6% (n = 44/59) with psychosis and in 80.9% (n = 72/89) with general mental distress. A high proportion of the sample reported having a persistent wish to die (41.8%), persistent suicidal thoughts (21.7%) or suicide attempt (14.8%) in the past month. As would be expected, gross self-neglect was a common presentation (Figure 3). However, neglect of common dangers was also high, affecting about 20% of those with psychosis. Self-injurious behaviour was noted in about 10% of participants. Exploratory analysis comparing those with and without psychotic disorders showed that those with psychosis were more likely to be older and to be on the streets for longer, while those without psychosis were more likely to attempt suicide and to be alcohol dependent (Table 3).

### Unmet needs in psychoses

**Figure 4:**
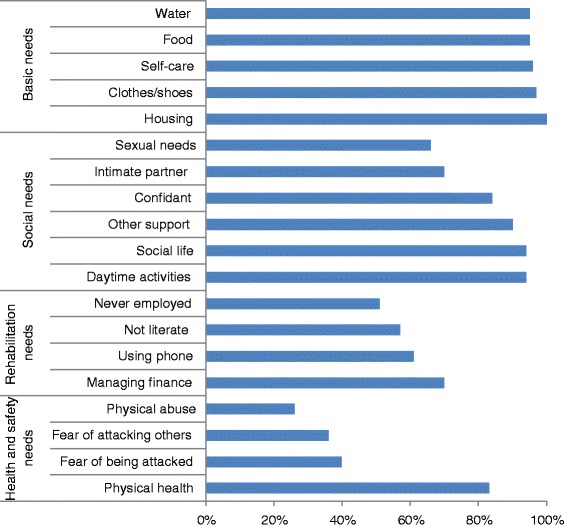
**Unmet needs of street homeless people with psychosis, Addis Ababa, Ethiopia.**

People with psychosis had extensive unmet needs in several domains. Detail on the magnitude of unmet needs is presented in Figure 4, and pertains only to people with psychosis who were able to provide information on a particular need domain. Therefore, the denominators varied, and are shown when relevant to interpretation. Basic needs, such as access to housing and access to adequate food and clean water, were unmet in 95% to 100% of the street homeless people with psychosis. The proportion with unmet needs in the social domain ranged from 66% (unmet sexual needs) to 94% (unmet social activity needs). Physical health needs were unmet in 84%. While 29.4% (n = 15/51) of respondents had some form of disability, 8 of these 15 patients had significant physical impairments (visual and sensory impairment and impaired mobility). Based on staff observation or report from participants, 12% (9/75) were considered at immediate risk of self-harm or being exploited. About 19% (11/58) reported a history of imprisonment since becoming homeless. Six individuals reported a history of sexual abuse, of which three were women. A large proportion also had unmet needs in the areas of functioning and rehabilitation; for example, independent use of public services, managing finances and basic literacy skills.

Only 10.5% (6/57) of those with psychosis had ever received treatment. None of the respondents (n = 61) were receiving any support from their families. Similarly, a low proportion of respondents reported receiving support from churches (2/61), mosques (1/61), charity organizations (4/61) or the neighbourhood (3/61) to address these unmet needs.

### Causes of homelessness

Family reasons were the major reasons reported to lead to homelessness (41%; n = 25/61), and economic reasons were reported to be directly relevant to becoming homeless in about a third (36%) of cases with psychosis (Table 5). Mental illness and treatment seeking for mental illness were cited as the main reason for homelessness in only five cases (8%); however, mental illness is likely to have been relevant to the homelessness of those reporting family disagreements, and for those who claimed not to know the reason for their homelessness. If this assumption is correct, mental illness may have made some contribution to the homelessness of over half of the cases (n = 33/61).

**Table 5:** **Reasons for homelessness among individuals with psychoses**

**Reason for homelessness**	**Number**	**Percent**
Death of primary carer	5	8.2
Separation from family	2	3.3
Disagreement with family	15	24.6
Run away from home	3	4.9
Mental illness	3	4.9
In search of treatment	2	3.3
In search of a job or education	12	19.7
After leaving the army	3	4.9
Economic problem	8	13.1
No reason	2	3.3
Don’t know	6	9.8
Total	61	100.0

## Discussion

Our study demonstrates the high burden of mental disorder and unmet health and social care needs of the street homeless in Ethiopia. To our knowledge, this is the first study in Africa, or any other low income country, attempting to determine the prevalence of mental disorders among street homeless people, or to estimate the unmet needs of street homeless people with psychosis. This lack of data is not only due to lack of interest or prioritization, but also due to the difficulties of identifying and evaluating this population. The mobile lifestyle of the homeless individuals, the lack of satisfactory collateral information, the vulnerability of the group and the need for sensitivity make identification difficult. Because many have no clinical records, evaluation can also be challenging. In our sample, complete information was difficult to obtain for some outcome and risk data, including some basic demographic data such as age, education, employment and marital status. We attempted to minimize the impact of these challenges by using experienced, well-trained mental health professionals as interviewers, with close supervision by a psychiatrist. The use of community nurses working locally, who knew their community and homeless residents, as well as our close collaboration with local community leaders and the police enhanced our capacity to identify the target group.

### Prevalence of mental disorders

The prevalence of mental disorders was higher than anticipated, based on reports from studies in higher income countries. Considering milder mental distress and alcohol use disorders in addition to psychosis, about 90% of street homeless adults had some form of current mental disorder. In reports from high income countries, the prevalence of mental disorder is around 60% in the street homeless [11]. A study of homeless shelter users from Rio de Janeiro, Brazil [44], reported the 12-month prevalence of any mental disorder and major mental disorders to be 49% and 19%, respectively. The higher rate of mental disorder in our sample may be partly explained by our focus on the street homeless, who are known to have higher rates of mental disorder [11]. Additionally the sample was chronically homeless, a factor known to be associated with increased psychiatric morbidity [45]. This in turn may have led to a disproportionately higher representation of those with mental health needs [45,46]. However, in studies investigating those who are newly homeless, even higher rates of mental disorder have been documented [46,47]. Our study is likely to have missed mobile and recently homeless individuals. In conjunction with the two-stage evaluation, which may screen out some individuals with psychosis, we might have underestimated the prevalence of psychosis.

A striking finding was the high proportion of individuals who reported attempting suicide in the preceding month. Although detailed assessment on the specific attempts was not possible, the finding is indicative of the vulnerability of this population. A relatively high proportion of respondents were considered to be at immediate risk of self-harm or exploitation. It is also of note that those without psychotic disorders had higher rates of suicide attempt and alcohol dependence. It has been reported that people with depression (with psychosis) had a higher rate of suicide attempt than those with schizophrenia, although the latter had made more dangerous attempts [48]. Thus, the higher rate of suicide attempts among those with non-psychotic disorders may be a reflection of that trend. Lower reporting among those with psychotic disorder (mainly schizophrenia) may be also an important factor. Both under-reporting and the ability to access alcoholic drinks among those with psychosis in our homeless sample may also be important factors in the finding of lower alcohol dependence among our sample. Despite comprising a small percentage of participants (10%), women appeared to be much more vulnerable to exploitation, for example 3/6 women (50%) who responded reported sexual abuse. All these findings represent a substantial level of neglect of the street homeless population, particularly those with mental illness.

### Unmet needs in psychoses

The available data confirm the extensive level of unmet needs in multiple domains (basic needs, social needs, and health and safety needs) among street homeless individuals with psychosis. This finding was anticipated from the outset given the lack of an organized social welfare system in Ethiopia. However, the more alarming finding was the little support the homeless mentally ill participants obtained from their family, local and international organizations, and the community. This was against the anticipation of the research group, which believed that support from the community and charities would be widespread. The treatment gap for mental disorders was extremely high. Many had co-morbid untreated physical illness or physical disability, and had not accessed care.

Although the Constitution of Ethiopia (Article 41/5) obliges the state to ‘allocate resources to provide rehabilitation and assistance to the physically and mentally disabled’ [49], homeless mentally ill individuals have little access to such provisions. Moreover, homeless individuals with mental illness are excluded from the activities of the Addis Ababa City Administration to rehabilitate the homeless. The focus of the rehabilitation by the city administration is primarily development of economic capacity, and those with mental illness have not been able to benefit from these initiatives. Given the high prevalence of psychoses and other distressing symptoms, which are associated with serious functional impairment, any initiative to improve the lives of homeless people should also focus on their mental health needs. It is of note that there has been very little interest from international organizations to support homeless mentally ill individuals to date.

### Causes of homelessness

Exploring what caused people to become homeless in the first place is difficult, particularly for people with severe mental disorders and when using a cross-sectional design. A large-scale multi-country study of homelessness involving eight low and middle income countries (Peru, South Africa, Zimbabwe, Ghana, India, Bangladesh, Indonesia and China) identified two main related reasons for homelessness [5]: poverty and failure of the housing supply system. Although economic reasons seemed to play a major role in the causation of homelessness within our sample, there are indications that mental illness may be an important factor in the causation and maintenance of homelessness in many homeless people with psychoses. The lack of treatment and other support services is likely to increase the incidence and prevalence of homelessness among the mentally ill. It is also worth noting that the stress of homelessness and access to substances of abuse can increase the risk of mental illness, although this is difficult to establish. Another important factor was the failure of the family unit, as was proposed in other similar settings [27,28]. This is of particular relevance, given the vital role that the family plays in traditional African societies, where the state does not provide a social safety net or where the provision of such social safety net is disorganised. Most of the participants were not originally from Addis Ababa, but had migrated in after becoming homeless. This migration may have been triggered by the loss of traditional family networks and support from the community. Although it is often believed that traditional communities in low income countries are tolerant of mental illness, there are no concrete data supporting this belief. To the contrary, a study from rural Ethiopia indicated that there may be less tolerance of the seriously mentally ill in such communities. An excerpt from a focus group discussion expresses the fear of traditional rural communities: ‘*Mad people…would run to the town…(they) are better tolerated in town. People in town are not afraid that these people would burn their houses. They would not chase them away like we do, that is why they run to town*’ [50].

### Limitations

Studies of homeless mentally ill people are extremely challenging. Despite our attempts to optimise assessment through training, supervision and attempts at repeated assessments, missing information was a major problem. Thus, there were missing values in some domains, sometimes in up to 33% of cases. We attempted to minimize the impact of missing data by focusing the analyses and the presentation on relevant descriptive data rather than investigating complex associations. Another limitation of the study was the difficulty of evaluating more mobile and recently homeless people. Such a study would require a different methodological approach. Finally, we did not look at the unmet needs of homeless people without psychosis. We intentionally focused on the needs of the more severely mentally ill population, given the need to prioritize service provision.

## Conclusions

The study confirms the widespread nature of unmet needs of the street homeless in the setting of an urban low income country with characteristics that are comparable with many other low income settings. Rapid urbanization and urban development, restructuring, migration, substance use problems and disruption of family networks are common developments across most low income countries and are likely to increase year on year. Mental health should be considered central to any endeavour to improve the lives of homeless people [46]. The low level of treatment receipt in this homeless sample from Ethiopia may be a reflection of the overall low accessibility of mental health care in the country [26,51]. Thus, governmental plans to scale up mental health care for the general population may improve treatment receipt and, potentially, prevent the onset of homelessness among the mentally ill. In this study, the proportion of homeless people with mental illness requiring urgent admission was relatively small, making the provision of community treatment and outreach feasible. Once immediate mental health needs are met, homeless people with mental illness will be better positioned to benefit from the social and economic interventions occurring as part of the wider community development. Given the overall role of families, it is crucial to try to reconnect with family networks, as challenging as this may be. Finally, the study also indicates some areas of priority for intervention among the homeless. Those at higher risk of exploitation, particularly women, and those at higher risk of self-harm, have to be prioritized in any intervention planning. We were not able to identify the acutely homeless, who may benefit the most from interventions. Part of the lesson from the work that has been performed in high income countries is that the ‘low-cost, no-care solution’ [2] is not a good intervention model to follow for low income countries. These countries should develop models of intervention with social engagement and family re-integration at their heart. However, what model of care should be implemented for this population in a low income country has to be defined. In addition to the above propositions of prioritization, community engagement and re-integration with family and rehabilitation, prevention of homelessness by providing early intervention, strengthening family support for those with ill family members, addressing substance abuse, providing protection from exploitation, and carrying out planned urbanization and investment in the care of the homeless seem essential.

## Additional files

Additional file 1: Table S1. Summary of studies on homeless people in Africa. Additional file 2: CANSAS. Camberwell Assessment of Need Short Appraisal Schedule. Ethiopian English Adaptation.
