# Supplementary material for: Burden of mental disorders and unmet needs among street homeless people in Addis Ababa, Ethiopia
Source: BMC Med. 2014 Aug 20;12:138. doi: 10.1186/s12916-014-0138-x (PMC4147171; doi:10.1186/s12916-014-0138-x)
Supplement: Additional file 1: Table S1. — Summary of studies on homeless people in Africa. [file 12916_2014_138_MOESM1_ESM.docx]

Table S1 Summary of studies on homeless people in Africa

| Reference | Year | Setting | Subject area | Findings | |
| --- | --- | --- | --- | --- | --- |
| Sagbakken et al[^1^](#_ENREF_1) | 2013 | Ethiopia* | Ethics of directly observed therapy (DOT) for TB | DOT created extra burden to homeless | |
| Mandalazi et al[^2^](#_ENREF_2) | 2013 | Malawi | HIV and STI risk in street children.  Qualitative study (N=23) | Low knowledge and increased risk practices | |
| Mathebula et al[^3^](#_ENREF_3) | 2013 | South Africa | Street experience and service use of young adults. Qualitative study (N=10) | High level of poor health, public hostility, trauma and addiction. But service use was good. | |
| Abdu et al[^4^](#_ENREF_4) | 2013 | Nigeria | Physical health of people on street (N=65) | Hypertension, visual problems and trauma said to be common. | |
| Embleton et al[^5^](#_ENREF_5) | 2013 | Kenya | Barriers and facilitators of drug cessation among street children. Mixed methods (FGD=30 participants and Survey N=146) | Barriers   - Addiction - Peer influence - Coping - Poverty - Family | Facilitators   - Self-caring - Desire to stop - Family - Peer influence |
|  |  |  |  | Lifetime prevalence of drug use   - Overall-74%. - Glue-67%; alcohol 47%; mira 33%; marijuana 29%) | |
| Mthembu et al[^6^](#_ENREF_6) | 2012 | Rwanda | KAP study about HIV. Street children. N=83 (Female=43) | Average knowledge. Major misconceptions, for example about the transmission of HIV and availability of vaccine | |
| Onofa et al[^7^](#_ENREF_7) | 2012 | Nigeria | Comparison of treatment outcome of vagrants and non-vagrants. Inpatient setting | - Most vagrants had diagnosis of schizophrenia - Had much longer duration in hospital (122 days vs. 61 days) | |
| Swahn et al[^8^](#_ENREF_8) | 2012 | Uganda | Prevalence of suicide ideation and attempts in the youth (14-24 years of age) in the past year + substance use. N=457 | - Suicidal ideation: 30.6% - Suicide planning: 22.9% - Suicide attempt: 19.8% - Needed medical help for attempt: 11.9% - Any drug use: 13.8% - Any drunkenness: 32.6% | |
| Elkoussi et al[^9^](#_ENREF_9) | 2011 | Egypt | Prevalence of volatile substance use in street children. N=120 | Prevalence: 91% | |
| Nada et al | 2010 | Egypt | Violence, abuse, alcohol and drug abuse in youth (12-17) | - Harassment or abuse: 93% - Sexual abuse among girls: - Alexandria: 90% - Cairo: 53% - Drug abuse: 62% | |
| Owoaje et al[^10^](#_ENREF_10) | 2009 | Nigeria | Sexual risk behaviour among youth (age 15-24) | - Increased sexual risk behaviour reported - Factors were: male gender, older age and regular alcohol use | |
| Fajemilehin et al[^11^](#_ENREF_11) | 2007 | Nigeria | Reason for becoming homeless in elderly (N=16) | Communal feud, violence, conflict, lack of support | |
| Wutoh et al[^12^](#_ENREF_12) | 2006 | Ghana | Sexual risk behaviour in street children (N=100) | Physical and sexual abuse higher among females | |
| Ayaya et al[^13^](#_ENREF_13) | 2001 | Kenya | Health problem of street children. Type 1 (on street children) N=38; Type 2 (of the street) N=47 | - Type 1 - URTI: 12.1% - Skin problem: 50.9% - Overall “burden of disease”: 467/1000 - Type 2 - Overall prevalence of disease: 833/1000 | |
| Lalor[^14^](#_ENREF_14) | 1999 | Ethiopia | Sexual violence against street children | 44% raped  26% sexually attacked | |

*a comparative study of the impact of DOTs regimen on patients from Ethiopia (N=10) and Norway. Patients were monitored over six months. Socially disadvantaged groups, such as the homeless, were described the most to lose from the DOT. The exact number of the homeless not stated.

1. Sagbakken M, Frich JC, Bjune GA, Porter JD. Ethical aspects of directly observed treatment for tuberculosis: a cross-cultural comparison. BMC Med Ethics. 2013; 14: 25.

2. Mandalazi P, Banda C, Umar E. Street children's vulnerability to HIV and sexually transmitted infections in Malawian cities. Malawi Med J. 2013; 25: 1-4.

3. Mathebula SD, Ross E. Realizing or relinquishing rights? Homeless youth, their life on the streets and their knowledge and experience of health and social services in Hillbrow, South Africa. Soc Work Health Care. 2013; 52: 449-66.

4. Abdu L, Withers J, Habib AG, Mijinyawa MS, Yusef SM. Disease pattern and social needs of street people in the race course area of Kano, Nigeria. J Health Care Poor Underserved. 2013; 24: 97-105.

5. Embleton L, Atwoli L, Ayuku D, Braitstein P. The journey of addiction: barriers to and facilitators of drug use cessation among street children and youths in Western Kenya. PLoS One. 2013; 8: e53435.

6. Mthembu S, Ndateba I. Exploration of knowledge, attitudes and behaviours of street children on the prevention of HIV and AIDS in the Huye district, Rwanda. East Afr J Public Health. 2012; 9: 74-9.

7. Onofa L, Fatiregun AA, Fawole OI, Adebowale T. Comparison of clinical profiles and treatment outcomes between vagrant and non-vagrant mentally ill patients in a specialist neuropsychiatric hospital in Nigeria. Afr J Psychiatry (Johannesbg). 2012; 15: 189-92.

8. Swahn MH, Palmier JB, Kasirye R, Yao H. Correlates of suicide ideation and attempt among youth living in the slums of Kampala. Int J Environ Res Public Health. 2012; 9: 596-609.

9. Elkoussi A, Bakheet S. Volatile substance misuse among street children in Upper Egypt. Subst Use Misuse. 2011; 46 Suppl 1: 35-9.

10. Owoaje ET, Uchendu OC. Sexual risk behaviour of street youths in south west Nigeria. East Afr J Public Health. 2009; 6: 274-9.

11. Fajemilehin BR, Ayandiran EO, Salami KK. Elderly destitution in Ile-Ife community of Osun State, Nigeria. Int J Nurs Pract. 2007; 13: 161-5.

12. Wutoh AK, Kumoji EK, Xue Z, Campusano G, Wutoh RD, Ofosu JR. HIV knowledge and sexual risk behaviors of street children in Takoradi, Ghana. AIDS Behav. 2006; 10: 209-15.

13. Ayaya SO, Esamai FO. Health problems of street children in Eldoret, Kenya. East Afr Med J. 2001; 78: 624-9.

14. Lalor KJ. Street children: a comparative perspective. Child Abuse Negl. 1999; 23: 759-70.
