# Supplementary material for: Burden of mental disorders and unmet needs among street homeless people in Addis Ababa, Ethiopia
Source: BMC Med. 2014 Aug 20;12:138. doi: 10.1186/s12916-014-0138-x (PMC4147171; doi:10.1186/s12916-014-0138-x)
Supplement: Additional file 2: — CANSAS. Camberwell Assessment of Need Short Appraisal Schedule. Ethiopian English Adaptation. [file 12916_2014_138_MOESM2_ESM.pdf]

CANSAS Camberwell Assessment of Need Short Appraisal Schedule. Ethiopian-English Adaptation

Need Rating: 0 = No problem 1 = Met need 2 = Unmet need 9 = Not known

	Interviewed		
Who is being interviewed (U=User, S=Staff, C=Carer)?	U	S	C
1. Accommodation<br>*What kind of place do you live in?*			
2. Food<br>*Do you get enough to eat?*			
3. Water<br>*Do you get enough clean water for drinking and washing?*			
4. Looking after the home<br>*Are you able to look after your home?*			
5. Self-care<br>*Do you have problems keeping clean and tidy?*			
6. Daytime activities<br>*How do you spend your day?*			
7. Physical Health<br>*How well do you feel physically?*			
8. Psychotic symptoms<br>*Do you ever hear voices or have problems with your thoughts?*			
9. Information on condition and treatment<br>*Have you been given clear information about your medication?*			
10. Psychological distress<br>*Have you recently felt very sad or low?*			
11. Safety to self<br>*Do you ever have thoughts of harming yourself?*			
12. Safety from others<br>*Do you fear that you may be victimized by others?(e.g., beaten up, assaulted or robbed)*			
13. Safety to others<br>*Do you think you could be a danger to other people's safety?*			
14. Alcohol<br>*Does drinking cause you any problems?*			
15. Drugs<br>*Do you take any drugs that aren't prescribed?*			
16. Social life<br>*Are you happy with your social life?*			
17. Intimate relationships<br>*Do you have a partner?(e.g. girl or boyfriend, husband or wife)*			
18. Other close relationships<br>*Do you have a person who visits you or someone you can confide in?(e.g., a close friend or family member)*			
19. Sexual expression<br>*How is your sexual life?*			
20. Child care<br>*Do you have any children under 18?*			
21. Education<br>*Any difficulty in reading, writing or understanding the <u>language of the place you reside in</u>?*			
22. Telephone<br>*Do you know how to use a telephone?*			
23. Transport			

Do you use the bus or taxi?			
24. Money<br>*How do you find budgeting money?*			
25. Benefits/ Practical support from family<br>*Do you get support of any kind from family and friends?*			
26. Clothes/ Shoes<br>*Do you get enough clothes and shoes to*			
Met needs – Count the number of 1s in the column			
Unmet need – Count the number of 2s in the column			
Total number of needs – A + B =			
Total number of needs for original version: A+B (excluding items in red font)=<br>NEW ITEMS TO BE EXCLUDED ARE ITEMS 2,13,18,26			

The adult CAN was developed by Mike Slade, Graham Thornicroft and others at the Health Service and Population Research Department, Institute of Psychiatry, King's College London. Further information from www.iop.kcl.ac.uk/prism/can. This version was translated into Ethiopian English by Abebaw Fekadu and Charlotte Hanlon at the Department of Psychiatry, School of Medicine, Addis Ababa University and Institute of Psychiatry, King's College London
